# Personalized prescription feedback to reduce antibiotic overuse in primary care: rationale and design of a nationwide pragmatic randomized trial

**DOI:** 10.1186/s12879-016-1739-0

**Published:** 2016-08-17

**Authors:** Lars G. Hemkens, Ramon Saccilotto, Selene L. Reyes, Dominik Glinz, Thomas Zumbrunn, Oliver Grolimund, Viktoria Gloy, Heike Raatz, Andreas Widmer, Andreas Zeller, Heiner C. Bucher

**Affiliations:** 1 Basel Institute for Clinical Epidemiology and Biostatistics, Department of Clinical Research, University Hospital Basel, CH-4031 Basel, Switzerland; 2 Clinical Trial Unit, Department of Clinical Research, University Hospital Basel, Basel, Switzerland; 3 SASIS AG, Solothurn, Switzerland; 4 Division of Infectious Diseases and Hospital Hygiene, University Hospital Basel, Basel, Switzerland; 5 Centre for Primary Health Care, University of Basel, Basel, Switzerland

**Keywords:** Antibiotics, Primary care, Routinely collected health data, Prescription feedback

## Abstract

**Background:**

Antimicrobial resistance has become a serious worldwide public health problem and is associated with antibiotic overuses. Whether personalized prescription feedback to high antibiotic prescribers using routinely collected data can lower antibiotic use in the long run is unknown.

**Methods:**

We describe the design and rationale of a nationwide pragmatic randomized controlled trial enrolling 2900 primary care physicians in Switzerland with high antibiotic prescription rates based on national reimbursement claims data. About 1450 physicians receive quarterly postal and online antibiotic prescription feedback over 24 months allowing a comparison of the individual prescription rates with peers. Initially, they also receive evidence based treatment guidelines. The 1450 physicians in the control group receive no information. The primary outcome is the amount of antibiotics prescribed over a one year-period, measured as defined daily doses per 100 consultations. Other outcomes include the amount of antibiotics prescribed to specific age groups (<6, 6 to 18, 19 to 65, >65 years), to male and female patients, in addition to prescriptions of specific antibiotic groups. Further analyses address disease-specific quality indicators for outpatient antibiotic prescriptions, the acceptance of the intervention, and the impact on costs.

**Discussion:**

This trial investigates whether continuous personalized prescription feedback on a health system level using routinely collected health data reduces antibiotic overuse. The feasibility and applicability of a web-based interface for communication with primary care physicians is further assessed.

**Trial registration:**

ClinTrials.gov NCT01773824 (Date registered: August 24, 2012).

## Background

Antibiotic resistance is a serious threat of public health worldwide. The consumption of antibiotics in a population is directly correlated with emergence of antibiotic resistance [1–3]. Reduced antibiotic prescriptions in primary care have been shown to be associated with substantial reductions of antibiotic resistance [3–6]. The most important reason for physician contact and antibiotic prescriptions in primary care are acute respiratory tract infections which are primarily of viral origin [7]. Multiple approaches of stewardship programs to lower antibiotic use in primary care have been investigated and those targeting acute respiratory tract infections were recently systematically reviewed [8]. The best evidence for beneficial effects have specific clinic-based education interventions for physicians and patients/parents, point of care testing, electronic decision support systems, communication training and delayed prescribing strategies. Although audit and feedback strategies showed relatively large effects on improved prescribing across various medical fields [9], for antibiotic prescribing the evidence is scarce [8]. These strategies are less resource intense than others such as communication training or face-to-face education with academic detailing, which allows a system-wide large scale application.

We present a randomized controlled trial that uses routinely collected health data for a nationwide prescription feedback intervention. It aims to reduce antibiotic overuse in primary care at a large scale using data from reimbursement claims. This pragmatic intervention targets primary care physicians with the highest antibiotic prescription rates in Switzerland. Here we describe the rationale and design of this trial.

## Methods/Design

### Study design and objective

This is a pragmatic randomized controlled parallel group trial with 1:1 allocation ratio of primary care physicians with the highest countrywide antibiotic prescription rates in Switzerland to either routine feedback and peer-benchmarking of antibiotic prescription behavior or usual care as control.

The primary objective of this trial is to assess the effect of a continuous personalized prescription feedback to primary care physicians on the amount of prescribed antibiotics. Secondary objectives are to assess effects on disease-specific quality indicators for outpatient antibiotic prescriptions, the acceptance of the intervention, and the impact on costs.

### Study setting and participants

This is a nationwide study conducted in Switzerland, enrolling more than half of all registered primary care physicians treating patients insured by Swiss statutory health insurers.

### Inclusion and exclusion criteria

**Fig. 1:**
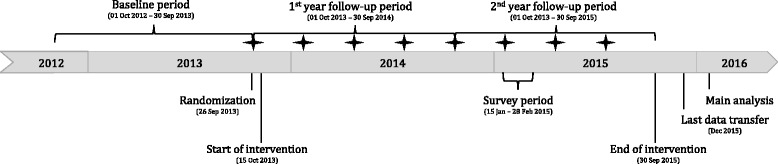
Timeline. Stars indicate feedback interventions by postal mail

There are only three selection criteria: (1) primary care physicians in Switzerland (identified using an individual central registration number, “Zentralregisternummer”, indicating board certified physicians) are included when they have (2) high antibiotic prescription rates (as defined by being among the 2900 top prescribers in Switzerland, see below). (3) Physicians with data available for only very few patients (i.e. <100 patients in a one-year period preceding the randomization) are excluded. Eligibility is determined based on data collected in the year prior to randomization (baseline; October 2012 to September 2013; Fig. 1).

### Intervention and control

**Fig. 2:**
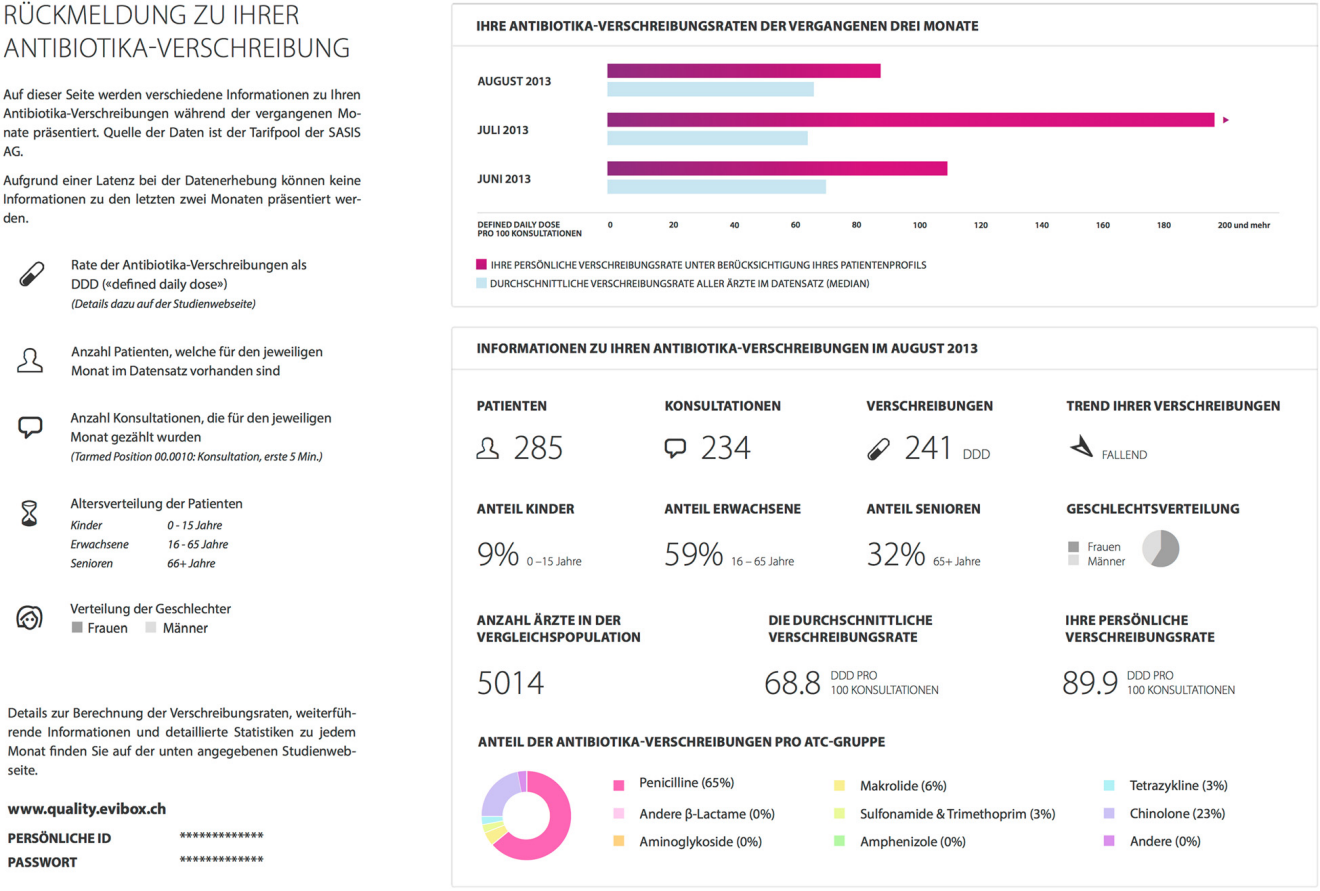
Example of Prescription Feedback in October 2013

The intervention starts in September 2013. In quarterly intervals (i.e. mid of January, April, July and September), a single-page overview including a continuously updated personalized prescription feedback is sent by postal mail to all physicians in the intervention group (example shown in Fig. 2). With the first mailing, physicians in the intervention group receive (1) an accompanying letter from the investigators providing basic information on the study and explaining the rationale of the intervention, which is described as quality improvement program, but no details about the conduct and design of the trial are provided; (2) a letter from SASIS AG (the organization providing the routinely collected data, see below) that clarifies privacy and data protection issues and justifies the rationale for the use the anonymized administrative data for scientific purposes; (3) evidence-based guidelines for optimized antibiotic use in primary care; (4) a response postcard allowing the physicians to opt-out from the intervention; (5) an individual access code to a dedicated study website with detailed prescription feedback and explanation of frequently asked questions on antibiotic use. Physicians in the control group receive no information.

We provide the information in three official languages (German, French, or Italian) according to the region where physicians are practicing in Switzerland. The information material, the feasibility and potential barriers of the intervention were pilot tested with primary care physicians attending a group session in June 2013. The evidence-based guidelines address the seven most important reasons for antibiotic prescriptions in primary care (acute unspecific upper respiratory tract infection, sore throat/acute tonsillitis/pharyngitis, acute rhinosinusitis, acute otitis media, acute bronchitis, community acquired pneumonia, and uncomplicated urinary tract infection). They were developed by LGH and HCB based on international evidence-based guidelines and Cochrane reviews. The guidelines were adapted to local conditions and reviewed by local experts in primary care, infectious diseases, pediatrics, and otolaryngology. The guidelines are provided to physicians in German or French.

There is no other change of concomitant care or practice. In July 2015, the last feedback is sent and the trial ends in September 2015, after 24 months of intervention.

### Outcomes

The primary outcome is the prescribed defined daily doses (DDD) of any type of antibiotics to any patient per 100 consultations (DDD/100c) over a one-year period, assessed for the first and second year of follow up.

Other outcomes are (1) change in the prescribed DDDs of all antibiotics per 100 consultations to young children (below 5 years), older children (6 to 18 years), adults (19 to 65 years), the elderly (above 65 years), to female or to male patients; (2) change in prescribed DDDs of specific antibiotic groups (i.e. tetracyclines (ATC J01A), amphenicols (ATC J01B), beta-lactams/penicillins (ATC J01C), other beta-lactams (ATC J01D), sulfonamides/trimethoprim (ATC J01E), macrolides/lincosamides/streptogramins (ATC J01F), aminoglycosides (ATC J01G), quinolones(ATC J01M), other antibacterials (ATC J01X), unspecified/unknown (ATC J01Z)) per 100 consultations (all patients). In sensitivity analyses, we use the crude number of antibiotic prescriptions per 100 consultations, to particularly address antibiotic prescriptions in children.

In ancillary analyses, we evaluate quality indicators of the European Surveillance of Antimicrobial Consumption (ESAC) for antibiotic prescriptions to assess the appropriateness of antibiotic prescribing practice for the seven common conditions that account for most antibiotic prescriptions in primary care, which are also addressed in the evidence-based treatment guidelines. We also plan to evaluate the usefulness and acceptance of the feedback intervention, and the impact on costs.

### Participant timeline

Figure 1 gives a schematic overview of the time schedule.

### Sample size

Our sample size calculation was based on a power of 90 % (1-beta) and a two-sided significance level of 0.05 (alpha) to detect an expected 5 % between group difference of antibiotic prescriptions per 100 patients over one year. We used a sample of routinely collected anonymous health data provided by the second largest Swiss statutory health insurer (Helsana). We applied a resampling approach (10,000 bootstrap samples) using a two-sided Wilcoxon rank-sum test for medians of prescriptions per 100 patients.

We assumed a 5 % reduction over 1 year in physicians who regularly access the online service (assuming this to be 30 % of the intervention group physicians), a 2 % reduction in the remaining physicians of the intervention group, and no reduction in the control group. The bootstrapping approach resulted in 1220 required participants per group. To separately assess effects only in those physicians who regularly access the online service, we required under the same assumptions 1427 physicians in the intervention group. With the intention to balance the groups at a 1:1 ratio the estimated sample size was 2900 participants, i.e. 1450 per group.

### Recruitment

All eligible primary care physicians identified in the dataset (see below) are ranked according to their prescription rates and we include the top 2900 prescribers. We had initially planned to include only physicians prescribing above the median. This would require at least 5800 primary care physicians registered in Switzerland to reach the target sample size of 2900. However, there were fewer registered physicians than expected, i.e. only 2484 physicians were above the median (77 DDD/100 consultations). Therefore, the 416 physicians having the next highest antibiotic prescription rate are additionally included.

### Treatment allocation process and blinding

All 2900 physicians selected from the database are randomly allocated (simple randomization, 1:1 ratio) to the intervention and control group by an independent researcher not further involved in the study and blinded to any physician data using R [10]. Perfect allocation concealment is ensured as all eligible participants are enrolled and randomized in one step. Thus, it is impossible that knowledge of the randomization sequence creates selection bias.

Due to the nature of the intervention, participants in the intervention group are aware of the intervention, albeit they are not aware of being in a randomized controlled trial. The outcome assessment is (formally) blinded, because all study relevant data are routinely collected by automatic procedures and by individuals not involved in the study (e.g. health insurance personnel).

The randomization list is kept secure and accessible only to personnel responsible for creating the feedback intervention during the conduct of the study. Two independent statisticians are involved, one for the generation of the feedback intervention and another for the study results analyses.

### Data source and privacy

All data used for this study are provided by SASIS AG, Switzerland, a data warehouse company of Santésuisse, an umbrella organization of Swiss statutory health insurers. Over 60 Swiss health insurers provide SASIS AG with aggregated administrative claims data for drug prescriptions (SASIS Tarifpool) and health care services data of licensed physicians (SASIS Datenpool) from patients insured in the statutory health system (the linked dataset covers approximately 60 % of the Swiss population) [11]. This reimbursement database that is used to inform payer-provider negotiations for fees for services [11].

SASIS AG ensures full blinding of all investigators regarding the identity of physicians allocated to the intervention and control groups. Only the involved statisticians (SLR, TZ) and health informaticians (RS) have access to the anonymous final dataset. All information material for physicians in the intervention group is prepared by use of anonymous IDs provided by SASIS AG and enclosed in sealed, opaque envelopes. The sealed envelopes are then addressed in the premises of SASIS AG using printed address labels linking anonymous IDs and address information. All study material is packed in the offices of SASIS and mailed by SASIS AG.

### Data management

We use routinely collected data on the reimbursement of prescriptions of antibiotics and outpatient consultations, geographical information, and information whether a physician is self-dispensing.

There can be a time lag of several months up (technically up to years) between the drug prescription and the reimbursement database entry at SASIS due to data collection processes and administrative claims management. Three months after prescription, approximately 84 % of the prescribed drugs are recorded in the database (the coverage rates vary over the year). The datasets provided by SASIS AG contain for each drug prescription the date of the prescription and the date of billing/reimbursement (aggregated by month), and the provided data are current to the end of the preceding month. For example, the mailed prescription feedback from January 2014 is based on a data package provided by SASIS in December 2013 that covers data of prescriptions that were recorded up to November 2013 for reimbursement. These reimbursement data reflect 84 % of the prescribed antibiotics in August, 69 % in September, 43 % in October, and 9 % in November. In the next updated data package (provided in March 2014), 97 % of antibiotics prescribed in August are recorded in the dataset (97 % for September, 95 % for October, and 90 % for November). The feedback shows the individual prescriptions for three consecutive months with a one-month gap (e.g. intervention in October contains feedback for June, July, August; Fig. 2). The peer-comparison shown in the personalized feedback as benchmark was the personal prescription rate adjusted by a population-based linear regression model including adjustments for the patient-mix using data on age groups, sex, and comorbidities.

Since patient level data are not available, we are unable to determine which patient receives which prescription. We also have no data on diagnoses, hospitalizations or mortality because such data is not routinely provided by Swiss health care providers to health insurers. However, we use data on prescriptions for other drugs to estimate the patient-mix of individual physicians. This drug data allows us to categorize patients into several groups of comorbidities. An overview of the routinely collected data used in this study is given in Table 1.

**Table 1:** Description of routinely collected data

Aggregated patient data	
Physician Identifier	Anonymized number
Canton	Location of practice (Swiss cantons)
- Time period	Year and month of reimbursement/prescription^a^
◦ Age category	For years 0–5; 6–10; 11–15; 16–18; 19–20; 21–25; then in 5-year categories up to 120 years
◦ Gender	Male, female or missing
▪ Total number of patients	Total number of patients treated by this physician in this month (as defined by the identifier and time period) who are within the age and gender category and have claims in this month.
▪ Total number of consultations	Total number of consultations of this physician in this month (as defined by the identifier and time period) who are within the age and gender category and have claims in this month.
Aggregated prescription data	
Physician Identifier	Anonymized number
Canton^b^	Location of practice (Swiss cantons)
- Time period	Year and month of eimbursement/prescription^a^
◦ Drug identifier	5- or 7-digit number to uniquely describe a drug (provided for antibiotics only)
◦ ATC code	ATC code up to level 5 for a drug (provided for antibiotics and selected other drugs)
▪ Age category	For years 0–5; 6–10; 11–15; 16–18; 19–20; 21–25; then in 5-year categories up to 120 years
▪ Gender	Male, female or missing
• Total number of prescriptions	Total number of prescriptions (drug packages) by this physician in this month (as defined by the identifier and time period) who are within the age and gender category and have claims in this month.

All data are from the SASIS-AG-Tarifpool and SASIS-AG-Datenpool and cover the years 2013 to 2015

^a^Two separate records, organized either by prescription date or by reimbursement date

^b^Drug dispensing status derived by canton: self-dispensing are physicians from the cantons (official acronym) AI, AR, BL, GL, LU, NW, OW, SG, SH, SO, SZ, TG, UR, ZG, ZH. Non-self dispensing: FR, GE, JU, NE, VD, VS, AG, BS, TI. Mixture (physician can choose to self-dispense or not): BE, GR

#### Ancillary analyses

We plan to collect information about the use of the personalized study website by individual physicians. In the second year of the study, we plan to invite physicians in the intervention group who used the personalized study website to take part in a small online survey on the acceptance of the feedback system and to record the diagnosis and treatment of consecutive patients presenting with acute respiratory or urinary tract infections. We plan to analyze the outcomes (see below) including only these intervention group physicians who use the dedicated study website. We plan to evaluate the reported usefulness of the guidelines and feedback system and to analyze quality indicators of the European Surveillance of Antimicrobial Consumption (ESAC) for appropriate outpatient antibiotic prescriptions [12]. An overview of the actively collected data in the survey is given in Table 2.

**Table 2:** Description of actively collected data for 1 ancillary research projects

Physician related data	
Physician Identifier	Anonymized number (as for routinely collected data)
Patient data	
Consecutive patient number	1 to 44
Age category	0–15, 16–65 or 65+ years
Gender	Male, female
Consultation period	Month of consultation (January or February 2015)
Consultation	First consultation or follow-up consultation
Diagnosis data	
Clinical diagnosis (ICPC-2 codes)	- Common cold (R74)
	- Tonsillitis/pharyngitis (R76)
	- Acute rhinosinusitis (R75)
	- Acute otitis media (H71)
	- Acute bronchitis (R78)
	- Pneumonia, community acquired (R81)
	- Urinary infection (U71)
	- Exacerbation of COPD (R95)
	- Influenza (R80) (each item recorded as yes/no)
Diagnostic tests used	- Chest X-ray
	- Streptococcus group A culture or rapid test
	- Urine dipstick
	- C-reactive protein
	- Leukocyte blood count (each item recorded as yes/no)
Prescription data	
Prescription	- Penicillins
	- Other β-lactams
	- Aminoglycosides
	- Amoxicillin/Clavulanic acid,
	- Macrolides
	- Sulfonamides and trimethoprim
	- Tetracyclines
	- Quinolones
	- Amphenicoles
	- Other antibiotics
	- No antibiotics prescribed (each item recorded as yes/no)

### Data analysis

We use a linear mixed model for the main analysis. The model includes as fixed effects the randomized group (intervention or control); time (baseline period, 1^st^ year, 2^nd^ year of follow-up); an interaction of randomized group with time; the total number of consultations at baseline; medication dispensing status of the physician; patient-mix type treated by the physician based on major comorbidities as assessed from co-medication use at baseline. The physician identifier is included as random effect. All covariates are selected prior to unblinding the data. Analyses are conducted after log-transformation since we expect a skewed distribution of prescriptions. The intervention effect is estimated (in the log scale) by calculating the relative change of the between-group difference from baseline to each year after randomization (with 95 % CI). Missing data are not imputed.

All analyses are based on the intention-to-treat principle, i.e. all participants are analyzed in the group to which they are randomized. The main analysis includes all randomized physicians. We also conduct an “on-intervention” analysis which excludes physicians from the intervention group who opt out. The nature of the study makes intervention cross-over and withdrawal in the control group impossible. Therefore, this analysis is technically both per-protocol and as-treated analysis.

### Monitoring

There is no data monitoring committee due to the special nature of this trial and because all data are collected during routine care. We appoint an independent general practitioner who will serve as a guardian in case of patient or physician complaints or any safety concerns and who coordinates further action. Ethics committees have guaranteed access to all original and processed data and permission to audit the project at any time (access to non-anonymized data must be authorized by SASIS AG due to Swiss data protection legislation).

### Ethics and dissemination

The study protocol is approved by all ethic committees responsible for all 26 cantons of Switzerland. Pragmatic trials may raise methodological, organizational and ethical problems that may be different from more traditional clinical trials [13–15]. This is an investigator initiated pragmatic trial which, in principle, is conducted in accordance to the ethical principles stated in the Declaration of Helsinki or the International Conference on Harmonization (ICH) guidelines on good clinical practice, whichever represents the greater protection of the individual. We disseminate details about the trial and its results following the completion of trial.

The study is publicly funded by the Swiss National Science Foundation. The Basel Institute for Clinical Epidemiology and Biostatistics is supported by an unrestricted grant from Santésuisse, the umbrella organization of Swiss health insurers.

### Reporting

We consider the reporting guidelines SPIRIT (study protocols) [16], RECORD (routinely collected data) [17], CONSORT (randomized trials) [18], and the CONSORT extension for pragmatic trials [19].

## Discussion

To our knowledge this is the first long-term and nationwide trial designed to reduce antibiotic prescription by provision of continuous routine feedback and evidence-based guidelines to primary care physicians with high prescription rates. Using a nationwide database of drug prescriptions and health care services data, we have developed a strategy that allows providing prescription feedback to physicians guaranteeing anonymization of all prescription and aggregated patient data.

In this trial, the participating physicians are not informed about the full nature of the trial and not asked for informed consent. In addition, physicians in the control group are not informed about the intervention. However, we believe this approach is acceptable [14, 20] because the trial does not involve patients directly but targets their physicians’ behavior. Physicians are autonomous to prescribe antibiotics according to their own best judgment of optimal treatment for patients as there is no extrinsic pressure to change prescription practices. No data used in this study allow any inferences on the patient level. We believe that a disclosure of the complete trial design to participants would introduce a substantial risk of bias, reduce the generalizability of the findings and severely reduce the value of the research. We plan to inform all physicians about their inclusion into this trial and disseminate the trial findings following the completion of the study. The first step is this publication of the detailed trial design.

This trial maximizes external validity due to its pragmatic design using “real world” data and in parallel ensures high internal validity by using a randomized design, near-perfect allocation concealment and follow-up, and blinded outcome data collection. The results will inform future guidelines and systematic reviews on antibiotic stewardship strategies and will improve decision-making of public health specialists, health insurers, health policy makers, and general practitioners.

## Abbreviations

ATC, anatomical therapeutic chemical classification system; CI, confidence Interval; CONSORT, consolidated standards of reporting trials; DDD, defined daily dose; ESAC, European Surveillance of Antimicrobial Consumption; ICPC-2, international classification of primary care – 2^nd^ edition; RECORD, Reporting of studies Conducted using Observational Routinely-collected Data; SPIRIT, standard protocol items: recommendations for interventional trials
